# 1711. Prospective Environmental Surveillance of Floors in a Pediatric Emergency Department During a Respiratory Syncytial Virus Surge in Ottawa, Canada

**DOI:** 10.1093/ofid/ofad500.1544

**Published:** 2023-11-27

**Authors:** Nisha Thampi, Jason Moggridge, Engluy Khov, Aaron Hinz, Lucas Castellani, Evgueni Doukhanine, Laura Hug, Rees Kassen, Derek MacFadden, Caroline Nott, Alex Wong, Michael Fralick

**Affiliations:** CHEO, Ottawa, Ontario, Canada; Mt Sinai Hospital, Toronto, Ontario, Canada; CHEORI, Ottawa, Ontario, Canada; Carleton University, Ottawa, Ontario, Canada; Sault Area Hospital, Sault Ste Marie, Ontario, Canada; DNA Genotek Incorporated, Ottawa, Ontario, Canada, Ottawa, Ontario, Canada; University of Waterloo, Waterloo, Ontario, Canada; University of Ottawa, Ottawa, Ontario, Canada; The Ottawa Hospital Research Institute, Ottawa, Ontario, Canada; The Ottawa Hospital, Ottawa, Ontario, Canada; Carleton University, Ottawa, Ontario, Canada; University of Toronto Department of Medicine, Toronto, Ontario, Canada

## Abstract

**Background:**

Annual respiratory syncytial virus (RSV) epidemics disproportionately impact infants and young children. Clinical surveillance is often restricted to laboratory-confirmed hospitalizations. Built environmental surveillance through floor sampling may provide population-level insights into RSV transmission dynamics and inform public health action to blunt community outbreaks.

**Figure:**
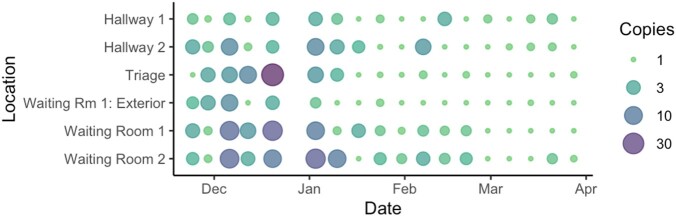
Quantification of viral detection from floor swabs in the pediatric emergency department by multiplex PCR

Each point represents the number of viral copies, plus one, detected from a swab captured at a given location (y-axis) on a given date (x-axis). A value of one copy indicates non-detection of RSV.

**Methods:**

A prospective environmental surveillance study was conducted in Ottawa, Canada from November 24, 2022 to March 24, 2023. Floors were swabbed in six areas of the emergency department (ED) of the sole pediatric acute care facility in Eastern Ontario. Samples were tested for RSV by a validated multiplex PCR assay. Floor swab positivity was compared to hospitalization data of patients with laboratory-confirmed RSV using Pearson’s correlation analysis to determine the degree of their association.

**Results:**

Over an 18-week period, 432 floor swabs were collected in the ED and 250 pediatric patients were hospitalized. RSV was detected in 65% of floor swabs, with more viral copies detected in congregate areas (waiting rooms, triage) (Figure). The correlation between swab positivity and RSV hospitalizations was 0.64 (95%CI 0.23-0.86).

**Conclusion:**

RSV was frequently detected on floors in the hospital-built environment and correlated somewhat with hospitalizations, likely reflecting community viral prevalence rather than severe RSV disease. Having real-time floor signals in different areas of the ED allowed for spatial resolution of the signal intensity, identifying higher RSV burden in areas where patients with infectious symptoms were more likely to congregate for prolonged periods of time. Floor sampling may be useful in developing spatially-refined approaches to infection prevention and control measures, particularly in indoor areas with increased respiratory viral transmission risks.

**Disclosures:**

**Evgueni Doukhanine, MSc**, DNA Genotek: DNA Genotek provided sampling swabs in-kind for this study in an unrestricted fashion. **Michael Fralick, MD**, ProofDx: Advisor/Consultant

